# Radiation Therapy for Malignant Lumbosacral Plexopathy: A Case Series

**DOI:** 10.7759/cureus.20939

**Published:** 2022-01-04

**Authors:** Naoko Sanuki, Shuji Kodama, Hidetoshi Seta, Mikiko Sakai, Hideki Watanabe

**Affiliations:** 1 Department of Radiology, Yokkaichi Municipal Hospital, Yokkaichi, JPN; 2 Department of Radiology, Mie Prefectural General Medical Center, Yokkaichi, JPN; 3 Department of Respiratory Medicine, Mie Prefectural General Medical Center, Yokkaichi, JPN; 4 Department of Nursing, Mie Prefectural General Medical Center, Yokkaichi, JPN; 5 Department of Nursing, Suzuka University of Medical Science, Suzuka, JPN

**Keywords:** malignant lumbosacral plexopathy, malignant psoas syndrome, pain management, palliative care, radiation therapy

## Abstract

Background

Malignant lumbosacral plexopathy is caused by a direct extension of an intrapelvic malignancy to involve the plexus nerves. In this report, we describe the effect of radiotherapy on patients with malignant lumbosacral plexopathy.

Patients and methods

We performed a retrospective review of the medical records of patients who underwent radiation therapy for pain caused by malignant lumbosacral plexopathy between 2017 and 2020 at our institution. The pain was measured using a numeric rating scale (0-10) at initiation and completion of radiotherapy or at the time when the maximum response was observed.

Results

A total of 12 tumor sites in 11 patients were included. Eight of the tumors invaded the iliopsoas muscle, and the remaining four invaded or abutted the piriformis muscle. The mean duration of follow-up was 215 days (31-675 days). All patients achieved pain relief at the end of radiotherapy, with complete resolution of pain in nine patients. The maximum effect was seen at a mean of three weeks (1-12 weeks) after the initiation of radiotherapy. Toxicities related to radiotherapy included grade 1 diarrhea in four patients and grade 1 frequent urination in one patient. Two patients experienced a relapse of pain at one and two months, respectively, after achieving their maximal response.

Conclusion

Radiotherapy provides significant pain relief for patients with the malignant lumbosacral syndrome. The recognition and diagnosis of this syndrome, and the use of radiation therapy as a therapeutic option, are important. Patients should be offered all possible therapies, regardless of curative or palliative intent.

## Introduction

More than two-thirds of patients with advanced malignant disease experience pain. However, the rate of pain relief is still suboptimal, and undertreatment of cancer pain remains a challenging issue. Cancer pain can represent a variety of types of pain, including bone pain, somatic pain, and neuropathic pain involving the peripheral nervous system. One type of neuropathic pain, “malignant psoas syndrome,” was defined in 1990 by Stevens et al. [1]. This syndrome involves the nerve bundles in the lumbosacral plexuses and psoas muscles and is categorized as a subgroup of malignant upper lumbosacral plexopathy. The pain is difficult to control due to its dual nature: nociceptive and neuropathic. A similar type of pain is caused by damage to other muscles or nerves in the pelvis. For example, if a tumor involves the piriformis muscle or its nerve supply, it may cause intractable pain by a mechanism similar to that of malignant psoas syndrome.

These pain syndromes, despite differences in site of origin, are often seen in patients with pelvic tumors. In this report, we present our experience with 11 patients (12 pain sites) with the malignant lumbosacral syndrome who were treated with palliative radiation and discuss the role of the multidisciplinary approach.

## Materials and methods

Patients who underwent radiotherapy for pain caused by malignant lumbosacral plexopathy at our institution between April 2017 and December 2020 were included. The computed tomography-based radiotherapy treatment plans were generated for all patients. They were treated via a 10-MV photon beam, using the Clinac EX linear accelerator (Varian Medical Systems, Palo Alto, CA). Information from the medical records was retrospectively collected. The intent of treatment could be palliation of symptoms, local control, or both. Total doses and fractions were at the discretion of the radiation oncologist. The pain was measured using a numeric rating scale (NRS) of 1 to 10. We recorded the NRS score at the initiation of radiotherapy and completion of therapy. The time of the maximum response (defined as the lowest NRS) was also recorded weekly. A change of two or more in the NRS score was defined as "improvement." We also collected information on analgesic regimens and dosages. Adverse events were evaluated using Common Terminology Criteria for Adverse Events (CTCAE), version 4.0 [2].

Because this was a retrospective study, consent for study participation was not obtained from each patient. The study was conducted on an opt-out basis and approved by the ethical review board of our institution (2021-K1, approval number: 0-0073).

## Results

A total of 12 tumor sites in 11 patients qualified for study inclusion. The patient characteristics are shown in Table 1. Gynecological cancer was the most common etiology, followed by urological cancer and gastrointestinal cancer. A total of eight tumors invaded the iliopsoas muscle; the remaining four tumors invaded or abutted the piriformis muscle. The intent of radiotherapy was palliation for half and local control for the other half. The mean duration of follow-up was 215 days (31-675 days).

**Table 1:** List of patients F: female; Gy: Gray; LC: local control; M: male; NRS: numeric rating scale for pain; P: palliative care; RT: radiotherapy

Patient number	Primary cancer	Age & sex	Pain at presentation	Involved muscle	Intent of treatment	Dose (Grey)/number of fractions	NRS score before RT	NRS score at completion of RT	Weeks to maximum response	Analgesic use per day
1 (the first RT)	Ovarian cancer	49, F	Left groin and femoral region; inability to lie supine	Psoas	P	39/13	9	0	3	Hydromorphone 6 mg Loxoprofen 180 mg
1 (the second RT)	Ovarian cancer	49, F	Right groin and femoral region	Psoas	P	39/13	8	0	3	Hydromorphone 6 mg Loxoprofen 18 0mg
2	Endometrial cancer	74, F	Left sciatic/buttock area	Piriformis	LC	54 /30	4	0	1	Acetaminophen 1000 mg
3	Cervical cancer	75, F	From right lateral thigh to lower back	Psoas	LC	59.4/33	3	0	4	Acetaminophen 1200 mg Pregabalin 150 mg
4	Urachal cancer	47, M	Left groin, exacerbated by straining	Psoas	P	60/30	8	3	4	Loxoprofen 18 0mg
5	Cervical cancer	54, F	From right lateral thigh to lower back	Psoas	LC	21/7	4	0	2	Hydromorphone 2 mg
6	Cervical cancer	44, F	Left groin/sciatic area	Piriformis	LC	59.4/33	5	3	12	Oxycodone 40 mg Acetaminophen 2400 mg Pregabalin 50 mg Celecoxib 400 mg
7	Ureteral cancer	61, M	Right pelvis and groin	Psoas	P	30/10	4	0	2	Oxycodone 30 mg Loxoprofen 180 mg
8	Rectal cancer	71, M	Left sciatic/buttock area	Piriformis	LC	54/30	3	0	2	Oxycodone 20 mg Acetaminophen 1500 mg Pregabalin 150 mg
9	Bladder cancer	70, M	Inability to extend the hip joint and to bend forward	Psoas	P	30/10	4	1	3	Morphine 20 mg Acetaminophen 1500 mg
10	Gastric cancer	73, F	Left groin and femoral region; inability to lie supine	Psoas	P	39/13	8	0	1	Oxycodone 20 mg Mirogabalin 10 mg
11	Bladder cancer	80, M	Left groin/sciatic area	Piriformis	P	30/10	8	0	1	Acetaminophen 1200 mg

At the end of radiotherapy, all patients achieved improvement in pain relief, with pain resolution (NRS 0) in nine patients. The analgesic dose was reduced in two patients, unchanged in six patients, and increased in four patients. The maximum effect was seen at a mean of three weeks (1-12 weeks) after initiation of radiotherapy. Of the nine patients for whom the duration of effect could be assessed, two patients (rectal cancer and urachal cancer) experienced a relapse of pain at one and two months, respectively, after their maximal response. The other seven of these patients experienced pain relief with a mean duration of 205 days (70-481 days). Grade 1 diarrhea associated with radiation was observed in four patients (able to be self-managed), and grade 1 frequent urination was observed in one patient who had ureteric ductal carcinoma. In the latter patient, it was difficult to determine whether the adverse effect was due to bladder invasion of the primary tumor or the radiation therapy. No other adverse effects were observed.

As of March 2021, a total of eight patients had died. The mean time from completion of radiotherapy to death was 125 days (31-260 days).

Case presentation

Patient 1

**Figure 1:**
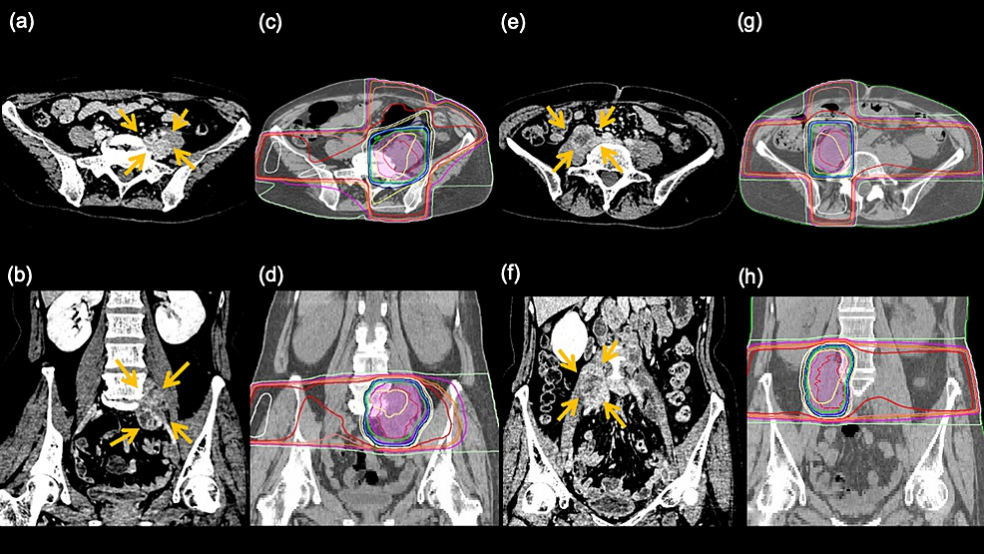
a 49-year-old woman with recurrent ovarian cancer (patient 1) The patient presents with bilateral involvement of the iliopsoas muscles. Palliative radiation is first administered to the left-sided tumor. (a) and (b): axial and coronal views of the tumor (arrows) on CT before irradiation. (c) and (d): radiation dose distribution with 95% isodose lines shown in green. Two weeks later, another course of radiation is performed for the right-sided tumor (e) to (h).

A 49-year-old woman with recurrent ovarian cancer after surgical treatment became refractory to chemotherapy and had enlarged lymph nodes invading the iliopsoas muscle on both sides. She was prescribed hydromorphone hydrochloride (6 mg/day) regularly, and as-needed, diclofenac sodium (25 mg), loxoprofen sodium hydrate (60 mg), and hydromorphone hydrochloride (4 mg) for analgesia. Despite this treatment, she was unable to lie supine due to pain and always slept in the lateral position, on her left side with her hip in flexion. When the patient’s case was discussed by the palliative care board, the radiation oncologist suggested palliative radiation therapy. A total of 39 Gray (Gy) in 13 fractions was administered to the painful left-sided tumors (Figures 1a-1d). At the time of treatment, it was difficult for the patient to be in a flat supine position; therefore, a custom-made device was created to fix the body so she could be treated in a position of hip flexion, with the upper body raised about 30 degrees. About 10 days after the initiation of radiation therapy, the patient was able to lie supine. Two weeks later, as the pain on her left side eased, the pain in the non-radiated right side became pronounced. Another 39 Gy of radiation was successfully provided to the right-sided tumors (Figures 1e-1h); this resulted in durable pain relief.

Patient 5

**Figure 2:**
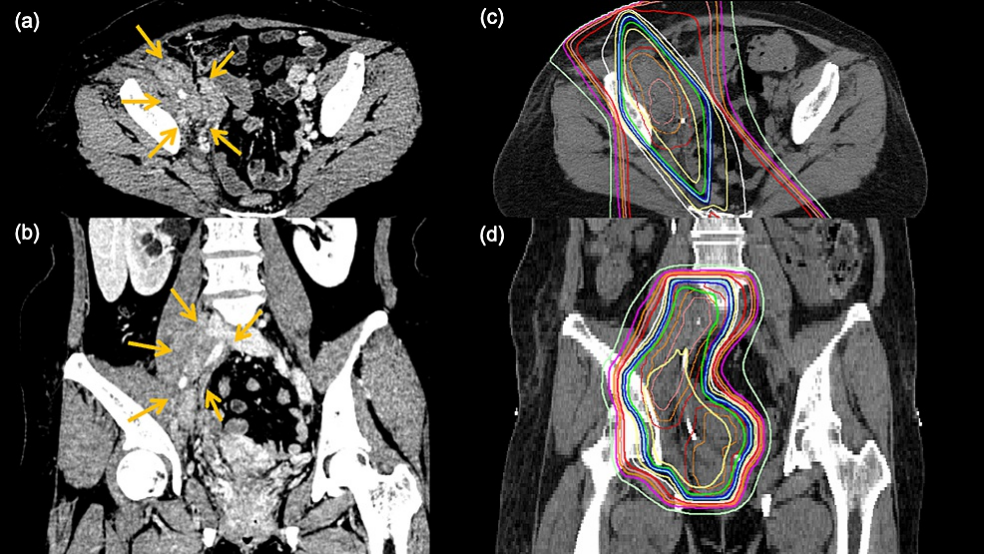
a 54-year-old woman with stage IV uterine cervix cancer (patient 5) (a) and (b): axial and coronal views of the tumor (arrows) on CT before irradiation. The numerous enlarged lymph nodes in the pelvis are infiltrating the right iliopsoas muscle (allows). (c) and (d): radiation dose distribution with 95% isodose lines shown in green.

A 54-year-old woman with stage IV uterine cervical cancer was admitted to the hospital with severe anemia, hydronephrosis, and difficulty walking due to pain radiating to her right leg. She had femoral vein thrombosis and edema in the right lower extremity and could not extend her right hip joint. On computed tomography, there were numerous enlarged lymph nodes in the pelvis in addition to the cervical tumor that infiltrated the right iliopsoas muscle. Hydromorphone hydrochloride (2 mg/day) was prescribed for analgesia with little effect. Her gynecologist consulted the radiation oncologist for palliative radiation, and 39 Gy over 13 fractions to the right pelvic lymph nodes was planned (Figure 2). The radiotherapy worked so quickly that her pain resolved after receiving only 12 Gy in four fractions; radiation was terminated at that time to prioritize chemotherapy. Approximately 17 months after completing radiotherapy, the patient is free of pain, and her lower-limb edema has resolved.

Patient 8

**Figure 3:**
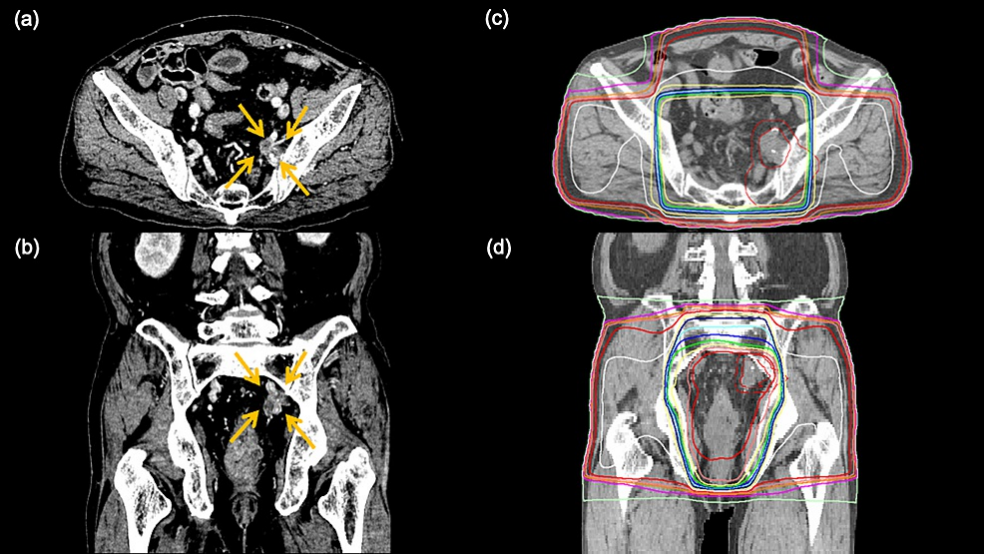
a 71-year-old man with locally advanced rectal cancer (patient 8) The patient undergoes semi-radical radiotherapy encompassing the primary tumor and metastatic pelvic lymph nodes. (a) and (b): axial and coronal views of the tumor (arrows) on CT before irradiation. One of the metastatic lymph nodes is in contact with the left ischiadic nerve (allows). (c) and (d): radiation dose distribution with 95% isodose lines shown in green.

A 71-year-old man with locally advanced rectal cancer was judged to be a poor candidate for radical treatment because of his comorbidities that included chronic heart failure, bronchial asthma, hypertension, alcoholism, and poor nutrition. Therefore, the decision to perform semi-radical radiotherapy alone was made. A total dose of 59.4 Gy in 33 fractions was planned, including the primary tumor and the metastatic lymph nodes in the pelvis. One of the metastatic lymph nodes was in contact with the left ischiadic nerve (Figure 3). At the start of radiation therapy, the patient complained of pain with an NRS score of 3, radiating from the left sciatic region to the left leg. Oxycodone hydrochloride hydrate (20 mg/day), acetaminophen (1500 mg/day), and pregabalin (150 mg/day) were prescribed for analgesia. About two weeks after the start of radiation therapy, his NRS score was 0. However, four months after the completion of radiation, the patient began to complain of recurrent pain in the left lower extremity, which required a higher dose of analgesic medication. On radiologic evaluation two months after radiation therapy, the size of the metastatic lymph node in the left pelvis had not changed significantly, while that of the primary lesion had decreased. Three months later, the metastatic lymph node had increased in size and had infiltrated the left piriformis muscle; this was thought to be the cause of his pain. In consideration of the cumulative gastrointestinal dose of radiation, repeat radiation was not offered.

## Discussion

Malignant lumbosacral plexopathy is caused by tumor involvement of the plexus nerves, itself resulting from direct extension from an intrapelvic malignancy [3]. The resulting pain is often described as aching, pressure-like, or stabbing; weeks to months later, the pain is followed by numbness, paresthesias, or weakness.

The condition is broadly but not exclusively classified by the site. Lower plexopathy is most common and occurs in over half of patients with malignant lumbosacral plexopathy [3]; the associated symptoms and signs are related to the L4 to S1 distribution. Pain can occur in the buttocks and perineum or can radiate to the posterolateral area of the thigh and leg. Upper plexopathy accounts for one-third of patients with lumbosacral plexopathy. Pain may be experienced in the back, lower part of the abdomen, flank, iliac crest, or the anterolateral portion of the thigh. A subgroup of these patients experiences malignant psoas syndrome, which is characterized by upper lumbosacral plexopathy, painful fixed flexion of the ipsilateral hip, and radiologic findings of malignant involvement of the ipsilateral psoas muscle [1].

All patients in this series had upper or lower malignant lumbosacral plexopathy, with contact or invasion of the tumor into the pelvic muscles, specifically, the iliopsoas or piriformis muscles. Although there is no malignant pain syndrome specific to the piriformis muscle, its involvement may be categorized as a lower lumbosacral plexopathy and explained as similar to piriformis muscle syndrome, with sciatica exacerbated by internal hip rotation. The tumor itself may produce symptoms regardless of hip rotation.

The pain associated with malignant psoas syndrome is usually difficult to treat. Its mechanism is proposed to be a combination of neuropathic pain from lumbosacral plexopathy and somatic nociceptive pain caused by muscle spasms due to tumor infiltration of the psoas muscle [1,4]. Multimodal medication is required, including opioid or nonopioid analgesics and adjuvant therapies, although the effect of analgesic agents is often limited. Radiation therapy has been occasionally adapted for both cancer-directed and palliative therapy, although its efficacy remains to be clarified with only limited case reports available [1,4-6]. Agar et al. [4] reported their four patients and reviewed the 23 case reports in the literature to describe the relevant clinical features and the use of multidisciplinary pain management. There is a couple of other case reports on radiotherapy for malignant psoas syndrome achieving durable pain relief [5,7]. Gelontopoulus et al. [8] reported their 26-year experience in the largest series of patients with malignant psoas syndrome treated with palliative radiotherapy. All 18 patients achieved significant pain relief and an improvement in overall health-related quality of life. The median duration of pain relief was approximately 3.5 months at a median of 5.5 months of follow-up. This was also true in our patients; some achieved even longer pain control. We found a median or mean duration of response of about 8.8 or 6.8 months, respectively, and it is likely that we included patients with more aggressive treatment intentions than the patients described in previous reports. This suggests that radiotherapy can maintain quality of life for a long period of time-perhaps even for the rest of the patient's life.

In the current series, radiotherapy demonstrated remarkable efficacy for pain caused by malignant lumbosacral plexopathy. It should be noted that the time to pain relief was short, regardless of the histology, suggesting a possibility that radiation had its effect by a different mechanism than simple tumor shrinkage. Although we originally expected a reduction in the volume of the tumors that we thought were causing pain, the tumor shrinkage and the pain relief appeared to be unrelated in patients whose post-treatment images were available. This may be similar to palliative radiation of bone, in which the release of physical pressure by tumor shrinkage may not necessarily be the mechanism of symptom palliation. On the other hand, for the two patients whose pain flared up after the initial radiation effect, the influence of the radiosensitivity of the original tissue may be relevant to the regrowth of the tumor.

Agar et al. [4] propose that malignant psoas syndrome comprises symptoms related to the degree of anatomical destruction, with associated inflammation and muscle spasm, and also to neuropathic pain caused by the lumbosacral plexopathy. Tsuchiyama [9] reported a patient with bilateral malignant psoas syndrome caused by advanced bladder cancer, with rapid progression of a severe crouching posture that was adopted to relieve pain. The patient died eight months following the onset of the disease. An autopsy revealed disseminated tumor spread, including to multiple lymph nodes in the retroperitoneal cavity that fused with and replaced the psoas major muscle, with few remnants of normal muscle fibers remaining. Although metastatic tumors involving skeletal muscle are rare, and few mechanisms have been identified that can be generalized, the postulated mechanisms include muscle motion and pH and lactic acid levels causing mechanical tumor destruction and inhibition of angiogenesis [10]. The poor and progressive nature of this syndrome may contribute to the prompt effects of radiation for pain relief, probably due to reasons other than tumor shrinkage itself.

Since radiation therapy is very effective, it is essential to offer this treatment to patients, even if they are experiencing flexion contractures. Our case series had three patients in whom the supine position was too painful to maintain, but all were successfully treated in various positions: hip flexion, forward bending, and supine with the upper body raised, using a customized body fixation device. All three patients experienced improved ability to conduct activities of daily life and were able to lie supine again and resume walking.

Lumbosacral plexopathy due to malignancy is considered to be a rare entity; its incidence in patients with cancer is reportedly 0.71% [11]. However, this low prevalence may reflect significant under-recognition of the condition [1,3,6]. The subjects of the present study of malignant lumbosacral plexopathy were diverse and not confined to malignant psoas syndrome. The purpose of radiation therapy ranged from palliation, under a best supportive care regimen, to semi-radical treatment for locally advanced symptomatic disease. It is important to note that our series of patients undergoing radiotherapy for malignant lumbosacral plexopathy also accounts for less than 1% of all patients with a diagnosis of malignancy each year. There could potentially be many more patients who could benefit from radiation therapy. At our institution, patients who may need palliative care intervention are routinely listed and discussed at the palliative care board. Our case series reaffirms the importance of a multidisciplinary board as well as of imaging evaluation to assess the cause of symptoms, even in patients who are prescribed the best supportive care regimens. Cross-sectional imaging, such as computed tomography, is crucial for evaluating lumbosacral plexopathy.

The limitations of our study include the fact that it is a single-center, retrospective report and that detailed neurologic findings (e.g., a specific level of involvement, pain, and paresthesia) were not obtained. Nevertheless, this is the first report to describe the effect of radiotherapy for malignant lumbosacral plexopathy in a wide range of eligible patients. There remain many issues that need to be discussed in the future, including increased awareness of this entity, proper diagnosis, adequate medical treatment, and the optimal dose and fractions of radiotherapy.

## Conclusions

Radiotherapy provides significant pain relief for patients with the malignant lumbosacral syndrome. The recognition and diagnosis of this syndrome and the offering of radiotherapy as a therapeutic option will have a significant impact on patients’ quality of life. It is important to allow patients to receive all possible therapies, regardless of curative or palliative intent.
